# Stigmatizing attitudes and low levels of knowledge but high willingness to participate in HIV management: A community-based survey of pharmacies in Pune, India

**DOI:** 10.1186/1471-2458-10-517

**Published:** 2010-08-27

**Authors:** Amita Gupta, Suvarna S Sane, Ajay Gurbani, Robert C Bollinger, Sanjay M Mehendale, Sheela V Godbole

**Affiliations:** 1 Johns Hopkins School of Medicine, Johns Hopkins University, Phipps 540, 600 N. Wolfe Street, Baltimore, Maryland 21287, USA; 2 Johns Hopkins Bloomberg School of Public Health, Johns Hopkins University, 615 N. Wolfe Street, Baltimore, Maryland 21205, USA; 3 Department of Epidemiology and Biostatistics, National AIDS Research Institute, Plot 73, Block G MIDC Complex, Bhosari, Pune, 411026, India; 4 David Geffen School of Medicine, University of California Los Angeles, 10833 Le Conte Avenue, Mailbox 221, Los Angeles, CA 90095, USA

## Abstract

**Background:**

The World Health Organization (WHO) recommends that the role of pharmacists in low-income settings be expanded to address the increasing complexity of HIV antiretroviral (ARV) and co-infection drug regimens. However, in many such settings including in India, many pharmacists and pharmacy workers are often neither well trained nor aware of the intricacies of HIV treatment. The aims of our study were; to determine the availability of ARVs, provision of ARVs, knowledge about ARVs, attitudes towards HIV-infected persons and self-perceived need for training among community-based pharmacies in an urban area of India.

**Methods:**

We performed a survey of randomly selected, community-based pharmacies located in Pune, India, in 2004-2005 to determine the availability of ARVs at these pharmacies, how they were providing ARVs and their self-perceived need for training. We also assessed knowledge, attitudes and perceptions on HIV and ARVs and factors associated with stocking ARVs.

**Results:**

Of 207 pharmacies included in the survey, 200 (96.6%) were single, private establishments. Seventy-three (35.3%) pharmacies stocked ARVs and 38 (18.4%) ordered ARVs upon request. The reported median number of ARV pills that patients bought at one time was 30, a two week supply of ARVs (range: 3-240 pills). Six (2.9%) pharmacy respondents reported selling non-allopathic medicines (i.e. Ayurvedic, homeopathy) for HIV. Ninety (44.2%) pharmacy respondents knew that ARVs cannot cure HIV, with those stocking ARVs being more likely to respond correctly (60.3% vs. 34.8%, p = 0.001). Respondents of pharmacies which stocked ARVs were also more likely to believe it was a professional obligation to provide medications to HIV-infected persons (91.8% vs. 78.8%, p = 0.007) but they were also more likely to believe that HIV-infected persons are unable to adhere to their medicines (79.5% vs. 40.9%, p < 0.01). Knowledge of the most common side effects of nevirapine, abnormal liver enzyme profile and skin rash, was reported correctly by 8 (3.9%) and 23 (11.1%) respondents, respectively. Seven (3.4%) respondents reported that they had received special training on HIV, 3 (1.5%) reported receipt of special training on ART and 167 (80.7%) reported that they believed that pharmacy staff should get special training on ART.

**Conclusion:**

There is a high willingness to participate in HIV management among community-based pharmacies but there is a tremendous need for training on HIV therapies. Furthermore, stigmatizing attitudes towards HIV-infected persons persist and interventions to reduce stigma are needed, particularly among those that stock ARVs.

## Background

In HIV management, pharmacists in many high-income countries play an important role in working with other health care providers (HCP) to ensure quality care and treatment, including educating patients about medications, adherence counseling, and assessing drug-drug interactions[1,2]. This requires being up-to-date regarding HIV and antiretroviral therapy (ART). In contrast, in low-income settings, the role of pharmacists and pharmacies has traditionally been a point of dispersal of medicines. Many, including the World Health Organization (WHO), recommend that this role be expanded to address the increasing complexity of ART and co-infection drug regimens[3]. However, in these settings as in India, many pharmacists and pharmacy workers are often not well trained, yet engage in practices that extend beyond medicine dispensing, including providing inadequate advice about medications and ailments[4-6]. Furthermore, it is common for individuals in low-income settings to seek the advice of pharmacists and medicine shops first rather than HCP for the treatment of common ailments[7-9]. In the context of HIV/AIDS and TB, such practices are particularly problematic and are likely to contribute to increasing drug resistance and treatment failure in the community[10].

India has an estimated 2.5 million persons living with HIV and many are in need of ART[11]. HIV-infected patients in India can access ART either by self-paying for their care in the largely unregulated private health sector or, as of 2004, at select government hospitals where ART is now being provided for free under the National AIDS Control Programme[11]. Despite free ART, some patients continue to access private pharmacies for their ART, including second-line antiretroviral drugs (ARVs) such as protease inhibitors, which remain largely unavailable in most government programs. In 2005, there were 559,408 registered pharmacies throughout India, reflecting a pharmacist to population ratio of 1:1,840, which is better than the average ratio of 1:2,300 reported in high-income countries[12].

We surveyed community-based pharmacies in Pune, India, a region with a high prevalence of HIV according to national India HIV surveillance estimates; to determine the availability of ARVs, provision of ARVs, knowledge about ARVs, attitudes towards HIV-infected persons and self-perceived need for training. Such data are needed to guide the role and needs of pharmacies and pharmacists in HIV management in low-income countries such as India.

## Methods

### Survey sample

The study was approved by the National AIDS Research Institute (NARI) Ethics Committee and the Johns Hopkins University Institutional Review Board. A survey of licensed community-based pharmacies in Pune, India was conducted between December 2004 and April 2005. Pune was selected because it is a high HIV prevalent district (HIV prevalence >1% in antenatal women), has many providers who have an HIV practice and because at the time, the Government's free ARV access program had not yet been initiated and therefore ART was accessed privately through community-based pharmacies. In order to accomplish this survey, we used a combination of sampling approaches. First, Pune, which consists of 48 electoral wards, was divided into four geographic areas (North, South, East, West). One electoral ward from each of these four areas was randomly selected. Trained study staff conducted a door-to-door mapping of each of these four wards to identify all pharmacies that were currently in business. All pharmacies identified by this method were approached for interview. Secondly, a list of 670 licensed pharmacies across Pune city was obtained from the Pune Chemists and Pharmacists Association (PCPA). A random sample of the pharmacies from this list was also included in the survey. Seven (11.8%) of 59 pharmacies that were identified from the ward sampling were already on the sample selected from the PCPA list and therefore were excluded, leaving 52 potentially eligible pharmacies from the ward sampling. Pharmacies were first contacted by telephone and an appointment was set up for the survey among those pharmacies that were contactable and willing to participate. The survey was administered by trained personnel via a face-to-face interview in the local Marathi language. The survey instrument was developed in English and translated. The survey was piloted and minor changes were made to improve the flow and clarity of the survey. We excluded pharmacies whose owners/pharmacists refused participation or whose doors were closed/unavailable for three visits at three different time points.

### Survey Instrument and data collection

We collected information regarding the characteristics of the pharmacy and pharmacist, as well as respondents' knowledge and perceptions regarding sexually transmitted infections (STIs), HIV, and ART. The survey also focused on specific characteristics of ARVs, such as whether ARVs are stocked, specific ARVs most commonly stocked, and the number of prescriptions dispensed by a particular establishment.

HIV- and ART-specific knowledge of the respondent was assessed through self-assessed ability to name ARVs as well as the objective confirmation of this ability. A knowledge-specific section assessed respondents' ability to correctly identify the side effects and appropriate combinations of ARVs. An assessment of respondents' perceptions and attitudes toward STIs, HIV, and ART was also completed using 5-point Likert scale. Questions which gauged respondents' confidence in dispensing ARVs, training on HIV, beliefs regarding HIV patients, professional obligation to provide treatment, and patient's responsibility for their illness were included. (Additional file 1)

### Statistical Methods

Double data entry was performed and data were entered into Microsoft Access. The major outcome variables considered were 1) whether or not the pharmacy stocked ARVs and 2) pharmacist's self-assessed need for HIV/AIDS training. The independent variables examined in relation to both outcomes were whether or not the respondent could name ARVs correctly, and the knowledge and attitude/perceptions scores. Other independent variables such as the size of the establishment, the presence of an air conditioner, and the number of years in business were also considered.

Size of the pharmacy was established based upon the number of prescriptions dispensed per month, with a small pharmacy being defined as less than 2000 and a large pharmacy as 2000 or more prescriptions dispensed per month. Correct naming of ARVs was first verified, and then a value was assigned to each respondent for correct number of drugs named. Respondents who correctly identified at least one ARV were then split into two groups based on whether they could name at least three drugs correctly or not. The items in the 5-point Likert scale were recoded into three categories: 'disagree', 'not sure', and 'agree'.

Knowledge scores were calculated using responses to six HIV-specific knowledge questions from the survey. Correct answers were given 1 point while incorrect responses or 'don't know' were coded as 0. Certain questions contained multiple parts, and points were awarded for responses from each part. Ultimately, assigned scores reflected a total possible range from a minimum of 0 to a maximum of 19, for all correct answers. Association of knowledge and attitudes with 'stocking ARV' was tested using Pearson's χ^2 ^test or Fisher's exact test when cell size was small (< 5 observations). Univariate and multivariate analyses were performed using logistic regression models. Odds ratios (OR) were calculated and the variables with p-value less than 0.2 in univariate were entered in the multivariate model to calculate adjusted odds ratios (AOR) and 95% confidence intervals, except for "correctly named ARV drugs" as it was highly collinear with "could name ARV drugs". Data were analyzed in SPSS 15.0 for Windows, SPSS Inc. 1989 - 2006.

## Results

### Characteristics of survey respondents

Of 309 pharmacy establishments approached, 207 (67.0%) consented to participation. Of these 207, 159 (76.8%) were sampled from the PCPA list of 670 pharmacies and 48 (23.2%) were sampled by approaching every single pharmacy mapped in the four electoral wards. The response rate for the ward-sampled pharmacies was 92.3% (48/52). The majority of the survey respondents were the owners of the pharmacy (n = 183, 88.4%). (Table 1) Of 207 pharmacies, 200 (96.6%) were single, private establishments, 4 (1.9%) were private pharmacies that were part of a chain, and 3 (1.5%) were hospital pharmacies (2 were part of the randomly selected sample from the PCPA list and one was identified from the door-to-door mapping).

**Table 1:** Characteristics of Pharmacy Survey Respondents in Pune, India*

Characteristic	N (%)
**Private Single Establishment**	200 (96.6)
**Position of respondent**	
Owner	183 (88.4)
Employee	24 (11.6)
**Utilities that are present on site**	
Refrigerator	207 (100.0)
Air Conditioner	26 (12.6)
Computer	96 (46.4)
Internet Access	20 (9.7)
Back-up Generator	74 (35.7)
**Median number of prescriptions filled per month (IQR)**	1500 (600, 2400)
**Size of pharmacy**	
Small (< 2000 prescriptions filled/month)	139 (67.1)
Large (≥2000 prescriptions filled/month)	68 (32.9)
**Pharmacist on site**	205 (99.0)
**Years in business**	
< 5 years	57 (27.5)
5 - 15 years	93 (45.0)
> 15 years	57 (27.5)
**Verification of authenticity of prescription**	
Never	10 (4.8)
Sometimes	37 (17.9)
Always	157 (75.8)
**Highest training of pharmacists at establishment (N = 175)**	
Diploma	147 (84.0)
Bachelors Degree	24 (13.7)
Post-graduate Degree	4 (2.3)
**Pharmacies that sell non-allopathic medicine for HIV**	6 (2.9)
**Pharmacies that stock ARV**	73 (35.3)
**Pharmacies that order upon request but do not stock**	38 (18.4)
**Median number of ARV prescriptions filled per month (range)**	2 (0, 100)
**Respondent could name at least one OI drug correctly (N = 206)**	134 (65.1)
**Respondents who said they could name ARV drugs**	134 (64.7)
**Pharmacists could actually name ARV drugs**	
At least one drug	131 (63.3)
At least three drugs	55 (26.6)
Five drugs	5 (2.4)

*N = 207 unless otherwise specified

The majority (67.1%) of these pharmacies were small establishments (dispensing less than 2,000 prescriptions per month), with a median number of one employee. For the majority of pharmacies, the highest training of on-site pharmacists was the diploma in pharmacy and only 4 (2.3%) had received any post-graduate training. Seventy-three (35.3%) pharmacies stocked ARVs. The most commonly stocked ARVs were lamivudine (n = 46, 63.0%), fixed- dose combination of stavudine/lamuvidine/nevirapine (n = 39, 53.4%), and zidovudine (n = 33, 45.2%). An additional 38 (18.3%) pharmacies ordered ARVs upon request. The reported median number of ARV tablets or capsules that patients bought from pharmacies at one time was 30, a two-week supply of ARVs (range: 3-240 pills). Of 111 respondents from pharmacies which either stocked or ordered ARVs upon request, 28 (25.7%) believed that their patients use the same prescription to refill ARVs without seeing a HCP. Additionally, 6 (2.9%) respondents reported that they sell non-allopathic medicines (i.e. Ayurvedic, homeopathy) for HIV.

### HIV and ART-related knowledge

Of 207 respondents, 134 (65.1%) were able to name at least one opportunistic infection (OI) drug correctly, 131 (63.3%) could name at least one ARV correctly and 5 (2.4%) could name 5 ARV drugs correctly. (Table 1) Ninety (44.2%) respondents reported knowing that ARVs can never completely cure HIV, with those stocking ARV being more likely to correctly respond (60.3% vs. 34.8%). (Table 2) Only a small proportion (≤15%) of respondents who stocked ARVs knew how many ARVs should be in an ideal ART regimen or that it was not safe to administer AZT with d4T, and only 1.4% of those who stocked ARVs were aware of efavirenz being a drug not recommended in early pregnancy.

**Table 2:** Responses to Knowledge Questions by ARV stocking status among 207 Survey Respondents in Pune, India

Question ("Correct answer")	Response	Stock ARV (n = 73)	Do not stock ARV (n = 132)	p-value*
**Minimum number of different drugs that should be included in an ideal antiretroviral treatment regimen **("3")	**Correct**	11 (15.1)	15 (11.4)	**0.007**
	**Incorrect**	46 (63.0)	59 (44.7)	
	**Don't know**	16 (21.9)	58 (43.9)	

**Antiretroviral drugs can completely cure HIV **("Never")	**Correct**	44 (60.3)	46 (34.8)	**0.001**
	**Incorrect**	12 (16.4)	22 (16.7)	
	**Don't know**	17 (23.3)	64 (48.5)	

**It is safe to administer AZT with d4T **("False")	**Correct**	8 (11.0)	4 (3.0)	**0.016**
	**Incorrect**	4 (5.5)	2 (1.5)	
	**Don't know**	61 (83.6)	126 (95.5)	

**Drug not recommended in pregnancy **("Efavirenz")	**Correct**	1 (1.4)	1 (0.8)	0.296
	**Incorrect**	8 (11.0)	7 (5.3)	
	**Don't know**	64 (87.7)	124 (93.9)	

*Association of 'Stocking ARV' with knowledge and attitude was tested by Pearson's χ^2 ^test or Fisher's exact test when cell sizes were less than 5.

When asked what advice is acceptable for a patient who cannot afford to take two tablets of a drug like 'Triomune' (fixed-dosed combination of stavudine/lamuvidine/nevirapine) a day, 72 (34.8%) answered that they would recommend speaking to a doctor right away. Knowledge of the two most common side effects of nevirapine, an abnormal liver enzyme profile and skin rash, were correctly reported by 8 (3.9%) and 23 (11.1%) respondents, respectively.

### Attitudes and perceptions

Fifty-four (26.1%) respondents felt that pharmacists or others in their establishment had a role in how patients take ARVs. Forty-six (22.2%) stated that they or others in their establishment had actually been asked by patients for advice on how to take ARVs, and 44 (21.3%) reported that they give advice to patients about taking ARV drugs. When asked to rate statements regarding their attitudes towards HIV patients, 171 (82.6%) respondents felt it was a professional obligation to provide medications to persons with HIV/AIDS and 197 (95.2%) reported that they did not worry about HIV exposure when dispensing medications to an HIV patient. Furthermore, 110 (53.1%) reported that patients with STIs/HIV have looser sexual morals, and 112 (54.1%) felt that HIV patients cannot adhere to ART. Respondents of pharmacies which stocked ARVs were more likely than those of pharmacies which did not stock ARVs to feel it was a professional obligation to provide medications to persons with HIV/AIDS (91.8% vs. 78.8%) but they were also more likely to feel that HIV patients are unable to adhere to ARVs (79.5% vs. 40.9%). (Table 3)

**Table 3:** Respondent Attitudes towards HIV Patients by ARV stocking status*

Statement	Response	Stock ARVs (n = 73)	Do not stock ARVs (n = 132)	p-value**
**"There is a professional obligation to provide medications to persons with HIV/AIDS"**	**Disagree**	6 (8.2)	12 (9.1)	**0.007**
	**Not sure**	0 (0)	16 (12.1)	
	**Agree**	67 (91.8)	104 (78.8)	

**"Patients with STIs/HIV are responsible for their illness" **(N = 206)	**Disagree**	12 (16.4)	16 (12.2)	0.184
	**Not sure**	12 (16.4)	36 (27.5)	
	**Agree**	49 (67.1)	79 (60.3)	

**"Patients with STIs/HIV have looser sexual morals"**	**Disagree**	6 (8.2)	23 (17.4)	0.079
	**Not sure**	21 (28.8)	45 (34.1)	
	**Agree**	46 (63.0)	64 (48.5)	

**"HIV patients cannot adhere to antiretroviral (ARV) regimens"**	**Disagree**	10 (13.7)	8 (6.1)	**< 0.01**
	**Not sure**	5 (6.8)	70 (53.0)	
	**Agree**	58 (79.5)	54 (40.9)	

**"Worry about HIV exposure when I dispense medicines to an HIV patient"**	**Disagree**	71 (97.3)	124 (93.9)	0.484
	**Not sure**	1 (1.4)	6 (4.5)	
	**Agree**	1 (1.4)	2 (1.5)	

*N = 207 unless otherwise specified

**Association of 'Stocking ARV' with attitudes was tested by Pearson's χ^2 ^test or Fisher's exact test when cell sizes were less than 5.

### Factors associated with ARV stocking and need for HIV training

Stocking of ARVs was associated with large pharmacy size, air conditioning, and respondents being able to name at least 3 ARVs. (Table 4)

**Table 4:** Association of Respondent Characteristics with the Stocking of ARV Drugs

Characteristics	Total N* (%)	Stock ARVs	Do not stock ARVs	Univariate OR (95% CI)	p-value	Multivariate AOR (95% CI)	p-value
**Air Conditioner**							
No	179 (87.3)	59 (80.8)	120 (90.9)	referent		referent	
Yes	26 (12.7)	14 (19.2)	12 (9.1)	2.37 (1.03 - 5.45)	0.042	3.77 (1.03 - 13.89)	**0.046**

**Years in Business**							
< = 5 years	57 (27.8)	14 (19.2)	43 (32.6)	referent		referent	
> 5 years	148 (72.2)	59 (80.8)	89 (67.4)	2.04 (1.02 - 4.05)	0.043	2.01 (0.83 - 4.90)	0.123

**Size of Establishment**							
Small	137 (66.8)	35 (47.9)	102 (77.3)	referent		referent	
Large	68 (33.2)	38 (52.1)	30 (22.7)	3.69 (2.00 - 6.82)	< 0.01	3.18 (1.39 - 7.24)	**0.006**

**Verification of Prescription Authenticity^# ^(**N = 202)							
Never	10 (4.9)	5 (7.0)	5 (3.8)	referent			
Sometimes	36 (17.8)	11 (15.5)	25 (19.1)	0.44 (0.11 - 1.84)	0.260	--	
Always	156 (77.2)	55 (77.5)	101 (77.1)	0.55 (0.15 - 1.96)	0.353		

**Could Name OI Drugs**							
No (Incorrect)	79 (38.5)	22 (30.1)	57 (43.2)	referent		referent	
Partially correct	30 (14.6)	12 (16.4)	18 (13.6)	1.73 (0.72 - 4.17)	0.224	0.83 (0.25 - 2.77)	0.762
Yes (Correct)	96 (46.8)	39 (53.4)	57 (43.2)	1.77 (0.94 - 3.36)	0.079	1.50 (0.61 - 3.70)	0.383

**Correctly Named ARV Drugs **(N = 131)							
Less than 3	76 (58.0)	33 (45.8)	43 (72.9)	referent		referent	
More than 3	55 (42.0)	39 (54.2)	16 (27.1)	3.18 (1.52 - 6.64)	0.002	3.54 (1.52 - 8.24)	**0.003**

**Knowledge Score^#^**							
Low	6 (2.9)	2 (2.7)	4 (3.0)	referent			
Middle	175 (85.4)	56 (76.7)	119 (90.2)	0.94 (0.17 - 5.29)	0.945	--	
High	24 (11.7)	15 (20.5)	9 (6.8)	3.33 (0.51 - 22.0)	0.211		

*N = 205 unless otherwise specified, # variables not included in multivariate model

In terms of HIV training, 7 (3.4%) respondents reported that they have received special training on HIV, 3 (1.5%) reported that they had received special training on ART specifically and 167 (80.7%) reported that they believed that people who work in pharmacies should get special training on ART. There was a trend that respondents with higher knowledge scores were more likely to think that pharmacy workers need ARV-specific training compared to those with low knowledge scores (adjusted OR 16.3, 95% CI 0.94-281.9, p = 0.055). (Table 5)

**Table 5:** Association of Respondent Characteristics with the Self-Assessed Need for Training

Characteristics	Total N* (%)	Self-Assessed Need for Training - Yes	Self-Assessed Need for Training - No	Univariate OR (95% CI)	p-value	Multivariate AOR (95% CI)	p-value
**Size of Establishment**							
Small	139 (67.1)	113 (67.7)	26 (65.0)	referent		referent	
Large	68 (32.9)	54 (32.3)	14 (35.0)	0.89 (0.43 - 1.83)	0.747	1.96 (0.75 - 5.13)	0.172

**Could Name OI Drugs**							
No (Incorrect)	79 (38.2)	68 (40.7)	11 (27.5)	referent		referent	
Partially correct	31 (15.0)	23 (13.8)	8 (20.0)	0.47 (0.17 - 1.3)	0.144	0.44 (0.11 - 1.7)	0.234
Yes (Correct)	97 (46.9)	76 (45.5)	21 (52.5)	0.59 (0.26 - 1.3)	0.189	0.57 (0.18 - 1.77)	0.330

**Could Name ARV Drugs**							
No	76 (36.7)	63 (37.7)	13 (32.5)	referent			
Yes (At least one)	131 (63.3)	104 (62.3)	27 (67.5)	0.79 (0.38 - 1.65)	0.539		

**Correctly Named ARV Drugs **(n = 131)							
Less than 3	76 (58.0)	61 (58.7)	15 (55.6)	referent		referent	
More than 3	55 (42.0)	43 (41.3)	12 (44.4)	0.88 (0.38 - 2.07)	0.771	0.60 (0.23 - 1.6)	0.307

**Knowledge Score**							
Low	6 (2.9)	3 (1.8)	3 (7.5)	referent		referent	
Middle	177 (85.5)	143 (85.3)	34 (85.0)	4.21 (0.81 - 21.76)	0.087	9.45 (0.76 - 117.7)	0.081
High	24 (11.6)	21 (12.6)	3 (7.5)	7.00 (0.94 - 52.04)	0.057	16.3 (0.94 - 281.9)	**0.055**

*N = 207 unless otherwise specified

## Discussion

Our study, based on a random sample of community-based pharmacies in an urban high HIV prevalence region of India, has several important findings. First, 35% of surveyed pharmacies stocked ARVs and an additional 18% ordered them upon individual requests, showing that at the time of our survey the treatment was relatively widely available in community-based pharmacies. Second, we identified a lack of adequate knowledge regarding HIV and ART among pharmacists, including among those who stocked ARVs. While not being able to list ARV names and side effects may have been expected amongst those respondents whose pharmacy did not stock ARVs, less than one-third knew that ARVs can never completely cure HIV. Furthermore, only 6% respondents were aware that it is inappropriate to administer AZT with d4T and only 1% knew that efavirenz was not recommended during pregnancy. This lack of awareness raises concern about the qualification and ability of the respondents to distribute ARVs, particularly if this distribution is linked with advice given to the patient. We therefore identified significant knowledge gaps and need for targeted training for pharmacies, particularly those dispensing ARVs in the community.

Encouragingly, we found in a majority of pharmacy respondents a sense of professional obligation to provide medication to persons with HIV/AIDS. In addition, 95% reported that they did not worry about HIV exposure when dispensing medications to an HIV-infected person. Therefore, there appears to be a willingness to care for and provide ARVs to HIV-infected persons. Nevertheless, stigma towards HIV patients remains high with nearly two thirds of respondents believing that patients are responsible for their illness and 54% reporting that HIV patients have looser sexual morals and cannot adhere to ARV regimens. Such provider-based stigma can be a significant barrier to health care seeking and treatment for HIV-infected patients, and is associated with reduced quality of life and health outcomes[13-15]. Therefore, there is continued need to combat stigmatizing attitudes of pharmacists and other HCP towards persons living with HIV/AIDS.

Despite the fact that only 26% of those surveyed felt that pharmacy staff play a role in how patients take ARV drugs, a majority (81%) felt pharmacy employees should receive specific training on HIV treatment. Only 3% of respondents stated they had ever received training on HIV and only 1% had training on ARV drugs specifically. As has been seen with pharmacists treating tuberculosis, there is a willingness to provide treatment and advice to patients despite not having formal training[10]. Training for pharmacists in India is needed to increase knowledge of safe practices and regimens, including drug names, side effects, and dosages, but also to dispel certain cultural notions that lead to stigma amongst treatment providers. The administration of an educational or training intervention would likely be best achieved through an alliance between the public and private sectors, as private providers have been shown to not follow the regulations put in place by the public sphere alone[4,16]. However whether incentives or mandatory regulation would be optimal to implement an effective training intervention needs to be investigated[4].

It appears to be common practice for pharmacists in India to sell loose medications and partial prescriptions [9]. In our study, we found 65% of respondents of pharmacies which stock ARVs have sold 10 or less ARV tablets or capsules to a patient at one time. While this is often done to accommodate the patient's financial ability to pay for drug, with HIV, such practices can be potentially dangerous and lead to unanticipated treatment interruptions and increase the risk of drug resistance, treatment failure and disease progression. Furthermore, 25% of respondents felt that some patients may be using the same prescription and not visiting their doctor regularly. Therefore, there is a need and opportunity for the pharmacy to serve as a check point to ensure that patients are receiving appropriate prescriptions, instructions about drug safety, and regular treatment from registered HCP. They should dispense drugs only after receiving a valid prescription written by an authorized physician. In addition, pharmacies can also serve as important checkpoints for medication adherence as pharmacy records of drug dispensing have been used effectively for estimating adherence to ART[17]. In high-income settings, almost all pharmacies have computerized databases, which greatly facilitate patient prescription tracking, appropriate drug combinations, and adherence monitoring[18]. While only 46% respondents reported having a computer on site and only 10% reported having internet access, the use of computers and internet is spreading rapidly in India. Therefore strategies using computerized pharmacy databases should be explored and incentivized in India, as these are likely to yield great benefits over time.

Our study had a few limitations. We likely had some degree of social desirability bias, which may have impacted responses related to pharmacy practices, such as the verification of prescriptions. Use of mock clients, who present symptoms and requests to pharmacists who are unaware that they are being examined could be a technique used for future studies[19]. We conducted the survey in an urban and peri-urban setting in a high HIV prevalence state of India during a time when less costly or free ART was not widely available. Therefore, our results may not necessarily be generalizable to other regions and settings in India, such as rural areas or states with lower prevalence, where there may be a decreased HIV awareness. Furthermore, our survey consisted of largely small, private pharmacies, so the results may not directly reflect the situation of very large or public hospital pharmacies. Nevertheless, the study was conducted on a sizable population and used a rigorous method of mapping and random sampling to obtain a representative sampling of community-based pharmacies in the Pune area. Since most of the ART scale-up in India since 2004 has occurred in the public government ART pharmacies, there may have been some changes in private pharmacy practices, however this would need to be confirmed. By and large, even with an expansion in the Government supported ART roll-out program in India since 2004, the accessibility to ART has not reached beyond 25% of those in need of ART [11] and some patients continue to obtain ARVs, including second line ARV drugs, from pharmacies such as those included in our study. Thus, our findings have a major relevance even today.

The coupling of patient support and counseling with the distribution of treatment in licensed pharmacies could provide a much needed resource, particularly in settings where stigma may affect patients' quality of life and willingness to access treatment. Such a model has already been used in India, with the conception of a pharmacy run by persons living with HIV/AIDS for the specific provision of ART at a subsidized cost. However such a model cannot be a solution for every pharmacy in India, which provides medicines for HIV or co-infections.

## Conclusions

This study emphasizes that approximately half of the community-based pharmacies surveyed that were based in a large peri-urban area of India dispense ARVs. However there was low knowledge about HIV therapies and stigmatizing attitudes towards HIV-infected patients, even among those who stocked ARVs. However encouragingly, there appears to be high willingness to participate in the provision of care for HIV-infected persons and a perceived need for focused training on HIV therapies. Before the role of the pharmacist in India is extended beyond the traditional task of dispensing medications, further training particularly in pharmacies that stock or order ARVs is needed[1,20,21]. Pharmacies can serve as an excellent checkpoint for ensuring appropriate HIV and co-infection therapies but only if there is appropriate training, knowledge and willingness to serve in this role.

## Competing interests

The authors declare that they have no competing interests.

## Authors' contributions

AG (JHU) and SVG designed the study, analyzed and interpreted the data and prepared the manuscript. SS performed all data analyses and assisted in the preparation of the manuscript. AG (UCLA) assisted in data analysis and manuscript preparation. RCB assisted in data interpretation and revised it critically for important intellectual content. SMM helped with study design, obtaining funding for the study and editing of the manuscript. SVG also oversaw the study conduct, data acquisition and obtained funding for the study. All authors commented on drafts, have read the revised manuscript, and have approved the final version.

## Pre-publication history

The pre-publication history for this paper can be accessed here:

http://www.biomedcentral.com/1471-2458/10/517/prepub

## Supplementary Material

Additional file 1 **Pharmacy survey questionnaire**. Click here for file
