# Supplementary material for: Stigmatizing attitudes and low levels of knowledge but high willingness to participate in HIV management: A community-based survey of pharmacies in Pune, India
Source: BMC Public Health. 2010 Aug 27;10:517. doi: 10.1186/1471-2458-10-517 (PMC2939646; doi:10.1186/1471-2458-10-517)
Supplement: Additional file 1 — Pharmacy survey questionnaire. [file 1471-2458-10-517-S1.DOC]

WHO IS THE BEST PERSON TO APPROACH WHO CAN ANSWER THIS SURVEY – SELECT THE PERSON WHO IS MOST KNOWLEDGEABLE ABOUT DISPENSING AND BUSINESS PRACTICES AT THE ESTABLISHMENT.

**We are conducting a survey to better understand how people access drugs for medical care especially for treatment and care of HIV infection. We would like to ask you a few questions about your establishment, how you stock medications, and how patients buy them. Your participation in this survey is voluntary and all the information you provide will be kept completely confidential. You do not have to answer any questions you do not want to. If you have any questions about this survey contact Dr. Sheela Godbole at 26128286.**

Are you willing to participate in this survey? 1 Yes Initial _________ 0 No

If yes, please proceed.

What is today’s date? (day/month/year) ______ /______ /______

dd mm yy

Time of Interview ___________

Interviewer Initials __________

DATA ENTRY LOG

|  | Date | Initials |
| --- | --- | --- |
| Entry #1 |  |  |
| Entry #2 |  |  |

# Type

1. How would you describe your establishment?

Private single establishment 1

Private Part of a chain 2

Attached to a Government Hospital 3

Attached to a Private/ Trust Hospital 4

Other_______________________________ 5

1. Do you have any of the following in your establishment?

(check all that apply)

|  | Y | N |  | Y | N |
| --- | --- | --- | --- | --- | --- |
| 2.1 Refrigerator | 1 | 0 | 2.6 Back-up Generator | 1 | 0 |
| 2.2 Freezer | 1 | 0 | 2.7 Inverter | 1 | 0 |
| 2.3 Computer | 1 | 0 | 2.8 Telephone | 1 | 0 |
| 2.4 Internet Access | 1 | 0 | 2.9 Patient Information sheets | 1 | 0 |
| 2.5 Air conditioner | 1 | 0 | 2.10 Other______________ | 1 | 0 |

# Characteristics

1. What is your position at this establishment?

Owner 1

Employee 2

Other ________________________________ 3

4. How many years has your establishment been in business?

< 1 year 1

1 -5 Years 2

5-10 Years 3

10-15 Years 4

> 15 Years 5

5. How many employees work at this establishment?____#

6. On an average how many prescriptions do you fill **per** **month** at this establishment?____#

7. Do you have a pharmacist on site?

Yes 1

No 0 *Skip to 11*

1. If Yes, how many pharmacists work at this establishment either part-time or fulltime (including yourself if you are a pharmacist)? _______ #

9. Is there a pharmacist always on site?

Yes 1

No 0

10. What is the highest training of the pharmacists on site at this establishment?

Diploma 1

Bachelors Degree 2

Post Graduate Degree 3

Other___________________________ 4

11. How do you dispense medications?

OTC and Prescriptions 1

By Prescription only 2

OTC only 3

Other 4

Describe Other_________________________________________

12. How often do you verify whether a prescription has been written by a licensed provider?

Never 1

Sometimes 2

Always 3

Other 4

Describe other________________________________________

13. Do patients ever ask you or your staff for medicines to treat STD symptoms like Pus discharge from genitalia, ulcers on genitalia?

Yes 1

No 0 Go to 17

14. If yes to above what do you do at such times?

_______________________________________________________

_______________________________________________________

15. Have you ever dispensed medicine to treat persons with STD symptoms OTC?

Yes 1

No 0 Go to 17

16. If yes, can you list them:

|  | Symptom | Drugs |
| --- | --- | --- |
|  |  |  |
|  |  |  |
|  |  |  |
|  |  |  |

17. Can you name any drugs used for treating or preventing HIV/AIDS related Opportunistic Infections?

*List as many as you can*

Drug1 _____________________________________________

Drug2 _____________________________________________

Drug3 _____________________________________________

Drug4 _____________________________________________

Drug5 _____________________________________________

## ARV specific

18. Can you name any drugs used to treat HIV specifically, these drugs are commonly known as antiretrovirals (ARV)?

Yes 1

No 0

Don’t know 9

19. If Yes, List as many as you can

Drug1 _____________________________________________

Drug2 _____________________________________________

Drug3 _____________________________________________

Drug4 _____________________________________________

Drug5 _____________________________________________

20. Do you stock any antiretroviral (ARV) medicines at your establishment?

Yes 1 *Go to 22*

No 0 *Go to 21*

Don’t know 9 *Go to 21*

21. If No, i.e. you do not stock ARVs, do you routinely order them from other places when a patient requests them?

Yes 1 *Go to 23*

No 0 *Go to 35*

Don’t know 9 *Go to 35*

22. If Yes, which antiretrovirals (ARV) and which brands do you most commonly stock? *(Please list all ARVs that are currently on stock at your establishment. If you need more room, write on back)*

|  | Drug | Company | Quantity per bottle or strip | Average Cost (Rs) | Shelf or Refrigerator? |
| --- | --- | --- | --- | --- | --- |
| 1 |  |  |  |  | 1 shelf 2 refrig |
| 2 |  |  |  |  | 1 shelf 2 refrig |
| 3 |  |  |  |  | 1 shelf 2 refrig |
| 4 |  |  |  |  | 1 shelf 2 refrig |
| 5 |  |  |  |  | 1 shelf 2 refrig |
| 6 |  |  |  |  | 1 shelf 2 refrig |

23. Which of antiretroviral (ARV) drugs are sold most by your establishment (*list up to three ARV drugs)*?

| No. | Drug | Company |
| --- | --- | --- |
| 1. |  |  |
| 2. |  |  |
| 3. |  |  |

24. How many prescriptions for antiretrovirals (ARV) on average are filled by your establishment in *(provide your best estimate if you are not exactly sure)*

A Week _______________ (#)

A Month_______________ (#)

In the last year__________ (#)

25. What is the **minimum** number of ARV tablets or capsules that a patient has EVER bought from you at one time?

______________ tablets / capsules **(INT: Circle tabs or caps)**

1. What is the **maximum** number of ARV tablets or capsules that a patient has EVER bought from you at one time?

______________ tablets / capsules **(INT: Circle tabs or caps)**

1. What is the **average** number of ARV tablets or capsules that patients buy from you at one time?

______________ tablets / capsules **(INT: Circle tabs or caps)**

1. How many patients are regular customers buying antiretrovirals (ARV) drugs from your pharmacy? (a regular customer is defined as someone who has bought drugs from their pharmacy at least 3 times in the past 1 year) ________

29. Do you think any of your patients use the same prescription to refill antiretrovirals (ARV) medicines (i.e., use the same prescriptions without seeing a physician)?

Yes 1

No 0 *Go to 31*

Don’t know 9  *Go to 31*

Refused 8 *Go to 31*

30. If Yes, what percentage of patients do you think do this?_____%

31. Have any of your patients tried to return antiretrovirals (ARV) drugs to your establishment?

Yes 1

No 0

Don’t know 9

Refused 8

32. Do you pass any discounts on antiretrovirals (ARV) drugs to your patients?

Yes 1

No 0

Don’t know 9

Refused 8

32.1 If *yes*, what enables you pass along a discount?

___________________________________________________

33. Do you stock antiretrovirals (ARV) drugs that have passed the date of expiry?

Yes 1

No 0

Don’t know 9

Refused 8

34. What do you do if antiretrovirals (ARV) drugs have expired?

35. Do you know which physicians routinely prescribe antiretrovirals (ARV) drugs in your area?

Yes 1

No 0 *Go to 37*

Don’t know 9 *Go to 37*

Refused 8 *Go to 37*

36. If YES, can you please list provider names and location?

| Practitioner Name | Location |
| --- | --- |
|  |  |
|  |  |
|  |  |
|  |  |

37. Do patients ever ask you or others in your establishment for advice about how to take their antiretrovirals (ARV) drugs?

Yes 1

No 0

38. Do you ever give advice to patients about how to take antiretrovirals (ARV) drugs?

Yes 1

No 0

39. Do you think pharmacists or others in your establishment have a role to play in how patients take antiretrovirals (ARV) drugs?

Yes 1

No 0 *Go to 41*

40. If YES: in what way, please describe?

## Non-allopathic

41. Do you sell any non-allopathic (i.e. Ayurvedic, homeopathy) medicines for HIV at your establishment?

Yes 1

No 0 *Skip to Perceptions*

42. If YES, which are most commonly sold at your establishment?

## Perceptions

43. How confident do you feel about dispensing antiretrovirals (ARV) drugs?

Very confident 1

Somewhat Confident 2

Not very confident 3

Not confident at all 4

Refused 8

44. Have you received any special training on HIV?

Yes 1

No 0

45. Have you received any special training on antiretrovirals (ARV) drugs specifically?

Yes 1

No 0

46. Do you think persons who work in pharmacies should get special training on antiretrovirals (ARV) drugs?

Yes 1

No 0

## Attitudes towards HIV patients

|  | Strongly Disagree | Disagree | Not sure | Agree | Strongly Agree |
| --- | --- | --- | --- | --- | --- |
| 47.1 I believe there is a professional obligation to provide medications to persons with HIV/AIDS | 1 | 2 | 3 | 4 | 5 |
| 47.2 I believe that patients with STIs/HIV are responsible for their illness | 1 | 2 | 3 | 4 | 5 |
| 47.3 I believe that patients with STIs/HIV have looser sexual morals | 1 | 2 | 3 | 4 | 5 |
| 47.4 Many HIV patients cannot adhere to antiretroviral (ARV) regimens | 1 | 2 | 3 | 4 | 5 |
| 47.5 I worry about HIV exposure when I dispense medicines to an HIV patient | 1 | 2 | 3 | 4 | 5 |

HIV/AIDS POLICY

48. Are you aware that the Government Of India plans to provide free antiretroviral treatment at select government centers from April 2004?

49. Do you have any specific views/ concerns regarding this plan?

50. Will you refer your HIV patients to government centers where free ART will be distributed?

1 Yes 0 No 3 Not sure yet

## Knowledge

We would now like to ask you a few questions about your general knowledge about antiretroviral drugs

51. What is the minimum number of different drugs that should be included in an ideal antiretroviral treatment regimen?

One 1

Two 2

Three 3

Four 4

Don’t Know 9

52. If a patient cannot afford to take two tablets of a drug like ‘triomune’ a day, it is OK to advise them to (Circle all that Apply)

|  |  | Yes | No |
| --- | --- | --- | --- |
| 52.1 | Take one pill a day | 1 | 0 |
| 52.2 | Take half a pill twice a day | 1 | 0 |
| 52.3 | Take 2 pills a day on alternate days | 1 | 0 |
| 52.4 | Not take it at all | 1 | 0 |
| 52.5 | Speak to their doctor right away | 1 | 0 |
| 52.6 | Don’t Know | 9 | 9 |

53. Antiretroviral drugs can completely cure HIV after

One Year 1

Two Years 2

Three 3

Never 4

Don’t Know 9

54. Which of the following are common side effects of Nevirapine

(Nevimune)

|  |  | Yes | No |
| --- | --- | --- | --- |
| 54.1 | Diarrhea | 1 | 0 |
| 54.2 | Skin rash | 1 | 0 |
| 54.3 | Fever | 1 | 0 |
| 54.4 | Abnormal liver enzyme profile | 1 | 0 |
| 54.5 | 1 and 4 | 1 | 0 |
| 54.6 | None of the above | 1 | 0 |
| 54.7 | Don’t know | 1 | 0 |

55. It is safe to administer AZT with d4T?

True 1

False 0

Don’t Know 9

56. Which of the following drugs is not recommended in Pregnancy:

Nevirapine 1

Efavirenz 2

Zidovudine (AZT) 3

Indinivir 4

Nelfinavir 5

Don’t Know 9

**Additional comments:**

**Thank you very much for your patience and time! The information you have provided is very informative and important.**

**Thank You!!!**
